# Addressing mental health in university students: a call for action

**DOI:** 10.3389/fpubh.2025.1614999

**Published:** 2025-06-18

**Authors:** Qiang Li, Jiayi Li, Yisheng Fan

**Affiliations:** ^1^Shuyang Hospital of Traditional Chinese Medicine, Jiangsu, China; ^2^Management School, Tianjin Normal University, Tianjin, China

**Keywords:** mental health, public health, university, student, policy

## Abstract

Mental health has been a growing concern in universities worldwide, with an increasing number of students experiencing psychological problems impacting their academic and personal performance. The transition to university life is often accompanied by an increase in stress, including the need for independence, academic pressure, and social adjustment. For many students, this period can exacerbate pre-existing mental health conditions or trigger new struggles. This perspective mainly focuses on the vital part of mental health in education, and calls for the indispensable role that universities should play in supporting their students’ psychological well-being and integrating mental health education into the academic environment. The future of mental health in universities lies in continued innovation, collaboration, and dedication to ensuring that every student has access to the resources they need to thrive academically and personally.

## Introduction

Mental health has been a growing concern in universities worldwide, with an increasing number of students experiencing psychological problems impacting their academic and personal performance (1). Consistent studies highlight that a significant proportion of university students are troubled by mental challenges such as anxiety, depression, and stress, with some researches estimating that as many as 12–50% students will experience at least one mental health issue during their time at university (2). These challenges not only hinder academic and daily lives but also may contribute to higher dropout rates.

The transition to university life is often accompanied by an increase in stress, including the need for independence, academic pressure, and social adjustment. For many students, this period can exacerbate pre-existing mental health conditions or trigger new struggles (3). Besides, studies have shown that there were significant increases in mental health concerns during the COVID-19 pandemic, with many students reporting difficulty in coping with remote learning, social distancing, and the overall uncertainty of the situation (4). Nowadays, the profound impact of mental well-being on student success makes it important for universities to provide comprehensive mental health support services. This perspective mainly focuses on the vital part of mental health in education, and calls for the indispensable role that universities should play in supporting their students’ psychological well-being and integrating mental health education into the academic environment.

## Mental health challenges faced by university students

Social adjustment is another significant challenge. For many students, university is their first experience living away from home, leading to feelings of isolation and loneliness. The lack of a family support can contribute to the development of anxiety and depressive symptoms (5). Furthermore, navigating new social networks and adjusting to diverse cultural environments can be overwhelming, particularly for international students who may face additional language barriers, cultural differences as well as homesickness (6).

Moreover, students with pre-existing mental health conditions, such as anxiety disorders, depression, or eating disorders, may find the university environment exacerbates their symptoms. According to World Health Organization World Mental health surveys, the 12-month prevalence of any mental disorder was 20.3% among college students and pre-matriculation disorders are associated with elevated odds of attrition (7). The increased stress and demands of university life can trigger or worsen these conditions, making it harder for students to seek help or maintain their well-being.

In addition to academic and social pressures, causes of stress during college life also include biological factors such as age and gender, as well as financial burden (8). To be specific, stress-related psychiatric disorders demonstrate a twofold higher prevalence among females relative to males (9). Mental health issues among students are not only common but also deeply impactful. Ultimately, the mental health challenges faced by university students are multifaceted, ranging from academic stress to social adjustment. These challenges underscore the need for universities to prioritize mental health support and create an environment where students feel empowered to seek help.

## Barriers to effective mental health support

Despite the growing recognition of mental health as a critical issue among university students, barriers exist preventing effective support from reaching those in need. These obstacles often result in students either not seeking help or receiving inadequate care, exacerbating their mental health struggles.

One of the most significant barriers is stigma (10). Despite advancements in mental health awareness, many students still feel ashamed or embarrassed about their struggles, leading them to avoid seeking help. The fear of being judged by peers, faculty, or future employers often outweighs the desire for support. This stigma is particularly pronounced in cultures where mental health is viewed as a sign of weakness or failure. As a result, students may choose to suffer in silence rather than address their issues, further isolating themselves and worsening their condition.

Another major barrier is the limited availability of mental health resources (11, 12). Many universities, especially those with large student populations, struggle to provide sufficient counseling services. Waiting times for appointments can be prohibitively long, sometimes extending for weeks, which delays the intervention needed for students to manage their mental health effectively. In some cases, students may not even have access to a counselor who specializes in the issues they face. This lack of timely and appropriate care can lead students to either abandon their efforts to seek help or turn to ineffective alternatives, such as self-medication or unhealthy coping mechanisms (13).

Moreover, university faculty and staff are often not adequately trained to recognize the signs of mental health issues in students. While many professors and administrators are well-intentioned, they may lack the expertise or resources to offer the support that students need. In some cases, faculty may not feel comfortable addressing mental health issues or may not be aware of the available campus resources. This gap in knowledge can result in missed opportunities for early intervention, leaving students to struggle without proper support.

Finally, there is a lack of mental health education within the university courses (14). While academic programs may focus on specific disciplines, few universities provide comprehensive training on mental health literacy for students and staff. Without this knowledge, students may not recognize the early warning signs of mental health issues, nor may they understand how to seek help effectively. In recent years, peer education is an approach growing in popularity across school, which may provide a potential solution to this problem (15).

Addressing these barriers requires a multifaceted approach. Universities must work to reduce stigma through education, increase funding for mental health services, and ensure that resources are accessible to all students. Additionally, faculty and staff training should be prioritized to equip them with the skills to identify and support students in need. Only by tackling these barriers head-on can universities ensure that mental health support is both effective and inclusive, providing students with the tools they need to thrive.

## Best practices and successful initiatives

In recent years, many universities have recognized the importance of addressing mental health issues and have implemented a range of innovative programs aimed at supporting students’ well-being. These initiatives reflect a broader understanding of mental health as an integral aspect of student success, not only for academic achievement but also for personal growth and resilience.

One of the most successful practices is the integration of mental health education into university curricula. Incorporating mental health awareness into academic programs equips students with the knowledge and skills to manage stress, recognize signs of mental illness, and develop healthy coping mechanisms. For instance, some universities offer mental health literacy courses that teach students about the biological, psychological, and social factors affecting mental health. These courses often include modules on stress management, emotional regulation, and seeking help, which provide students with practical tools for maintaining mental well-being. By normalizing conversations around mental health and fostering a proactive approach, these programs help reduce stigma and encourage students to take ownership of their mental health.

Another effective strategy has been the development of peer-led support programs (16). Peer mentoring has become a cornerstone of many universities’ mental health initiatives. Peer mentors, who are often trained students, act as first responders to their peers’ mental health needs, providing emotional support and guidance in navigating available services. These programs have been particularly successful because they offer a non-threatening and relatable avenue for students to seek help. Studies show that students are more likely to turn to their peers rather than formal counseling services, making peer support an invaluable resource in creating a culture of mental well-being (17). Peer-led support programs can also reduce stigma by offering empathetic assistance to each other (18).

Additionally, universities have increasingly turned to digital platforms to offer mental health support, providing students with more flexible and accessible options (19). Digital tools like mental health apps and online therapy sessions are gaining traction as convenient alternatives to traditional in-person counseling. For example, platforms are now integrated into some university wellness programs, allowing students to access therapy or mindfulness exercises from the comfort of their homes. These digital resources are particularly beneficial for students who may feel uncomfortable or unable to attend face-to-face sessions due to stigma, time constraints, or other logistical barriers. Of course, while these platforms offer convenience and accessibility, privacy concerns are a significant issue, as users may be hesitant to share personal information due to fears of data misuse.

In addition to these direct services, many universities have launched comprehensive wellness programs that emphasize the importance of holistic health (20). These initiatives often focus on balancing academic, social, and physical well-being through fitness programs, relaxation techniques, and stress-reduction activities like yoga or meditation (21). Engaging in physical activities such as basketball and music like rap, has been shown to reduce stress, improve mood, and promote emotional regulation by increasing the release of endorphins and other neurochemicals, providing a fun and engaging way to support mental health. Such programs promote a more balanced approach to student life, which can reduce the likelihood of mental health issues arising in the first place.

The key to the success of these initiatives lies in their adaptability and comprehensive nature. By combining educational, peer, digital, and professional support, universities can create a more resilient and mentally healthy student body. Moving forward, universities should continue to expand these successful programs, tailoring them to meet the unique needs of their diverse student populations while promoting mental health as a cornerstone of academic success.

## Conclusion

**Figure 1:**
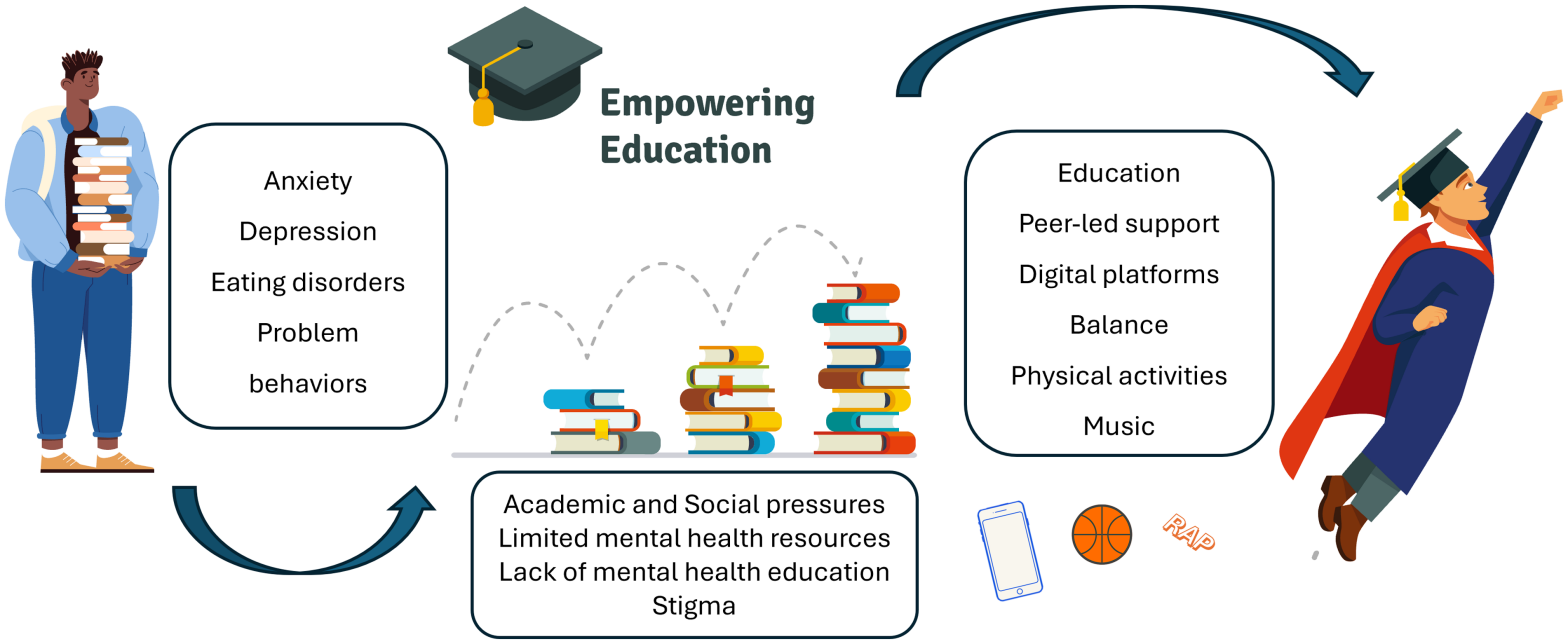
Challenge, barrier and best practice in addressing mental health in university students.

As universities continue to serve as vital parts for intellectual development and personal growth, the importance of mental health support has never been more pronounced. Mental health challenges among university students are not merely a byproduct of academic stress; they are complex issues that reflect broader societal pressures and individual factors. Addressing these challenges requires a comprehensive, multi-faceted approach are eagerly needed (Figure 1).

Universities have made significant strides in recent years, implementing progresses such as mental health literacy courses, peer-led support programs, digital mental health resources, and holistic wellness initiatives. These efforts have shown promising results in reducing stigma, improving student engagement with mental health services, and enhancing overall student well-being. However, much work remains to be done. Barriers such as stigma, resource limitations, and structural challenges still impede the effectiveness of mental health support in many institutions.

The future of mental health in universities lies in continued innovation, collaboration, and dedication to ensuring that every student has access to the resources they need to thrive academically and personally. Finally, this will contribute to the creation of a more supportive, resilient, and successful student population, with lasting positive effects on their academic and personal lives.

## Data Availability

The original contributions presented in the study are included in the article/supplementary material, further inquiries can be directed to the corresponding authors.
